# Diversity and Evolution of DNA Transposons Targeting Multicopy Small RNA Genes from Actinopterygian Fish

**DOI:** 10.3390/biology11020166

**Published:** 2022-01-20

**Authors:** Kenji K. Kojima

**Affiliations:** Genetic Information Research Institute, Cupertino, CA 95014, USA; kojima@girinst.org; Tel.: +1-650-961-4480

**Keywords:** *Dada*, DNA transposon, tRNA, 5S rRNA, domestication, dDada

## Abstract

**Simple Summary:**

DNA transposons are parasitic DNA segments that can move or duplicate themselves from one site to another in the genome. *Dada* is a unique group of DNA transposons, which specifically insert themselves into multicopy RNA genes such as transfer RNA (tRNA) genes or small nuclear RNA (snRNA) genes to avoid the disruption of single-copy functional genes. However, only a few *Dada* families have been characterized along with their target sequences. Here, vertebrate genomes were surveyed to characterize new *Dada* transposons, and over 120 *Dada* families were characterized from diverse fishes. They were classified into 12 groups with confirmed target specificities. Various tRNA genes, as well as 5S ribosomal RNA (rRNA) genes were inserted by *Dada* transposons. Phylogenetic analysis revealed that *Dada* transposons inserted in the same RNA genes are closely related. Phylogenetically related *Dada* transposons inserted in different RNA genes show the sequence similarity around their insertion sites, indicating *Dada* proteins recognize DNA nucleotide sequences to find their targets. Understanding how *Dada* discovers the targets would help develop target-specific insertions of foreign DNA segments.

**Abstract:**

*Dada* is a unique superfamily of DNA transposons, inserted specifically in multicopy RNA genes. The zebrafish genome harbors five families of *Dada* transposons, whose targets are U6 and U1 snRNA genes, and tRNA-Ala and tRNA-Leu genes. *Dada-U6*, which is inserted specifically in U6 snRNA genes, is found in four animal phyla, but other target-specific lineages have been reported only from one or two species. Here, vertebrate genomes and transcriptomes were surveyed to characterize *Dada* families with new target specificities, and over 120 *Dada* families were characterized from the genomes of actinopterygian fish. They were classified into 12 groups with confirmed target specificities. Newly characterized *Dada* families target tRNA genes for Asp, Asn, Arg, Gly, Lys, Ser, Tyr, and Val, and 5S rRNA genes. Targeted positions inside of tRNA genes are concentrated in two regions: around the anticodon and the A box of RNA polymerase III promoter. Phylogenetic analysis revealed the relationships among actinopterygian *Dada* families, and one domestication event in the common ancestor of carps and minnows belonging to Cyprinoidei, Cypriniformes. Sequences targeted by phylogenetically related *Dada* families show sequence similarities, indicating that the target specificity of *Dada* is accomplished through the recognition of primary nucleotide sequences.

## 1. Introduction

Transposable elements (TEs), also known as transposons or mobile DNA, include a wide variety of DNA segments that can, in a process called transposition, move or duplicate from one location in the genome to another [1,2,3]. Historically, eukaryotic TEs are divided into two classes: Class I and Class II, or retrotransposons and DNA transposons [4]. Retrotransposons include any autonomous transposons that encode a reverse transcriptase (RT) and non-autonomous transposons which transpose dependently on autonomous retrotransposons. Retrotransposons are further divided into several categories, long terminal repeat (LTR) retrotransposons, and non-LTR retrotransposons, often tyrosine recombinase (YR) retrotransposons (or *DIRS* retrotransposons) and *Penelope*-like elements (PLEs) as well [2,3,5,6,7,8]. DNA transposons known to date encode one of several types of “transposase” proteins. The DDD/E transposase is the most common transposase for both eukaryotic and prokaryotic DNA transposons [2,3,9,10]. In eukaryotes, two additional types of transposases are encoded by DNA transposons: tyrosine recombinase (YR) by *Cryptons* [11], and HUH nuclease by *Helitrons* [12]. 

Eukaryotic DNA transposons with DDD/E transposases are classified into 20 or so superfamilies [2,13]. All DDD/E transposases maintain three acidic residues, either DDD or DDE, at the catalytic center [9,14]. Besides these three acidic residues, some superfamilies have additional motifs, called signatures, in common. *Dada*, *hAT*, *Kolobok*, *MuDR*, and *P* share a C/DxxH motif between the second D and the last D/E residues in their transposases [9,14,15]. 

TEs are potentially harmful with the ability to reproduce and insert themselves into genes or other functional genomic sequences [16,17]. Some groups of TEs have evolved to minimize the chance for disrupting genes, through the targeted integration into certain types of repetitive sequences. Several different lineages of non-LTR retrotransposons have evolved to target various types of repetitive sequences, such as rRNA genes, transfer RNA (tRNA) genes, spliced leader exons, small nuclear RNA (snRNA) genes, microsatellites, telomeric repeats, or other TEs [18,19,20,21,22,23,24]. The endonucleases encoded by these non-LTR retrotransposons cleave the DNA specifically at the sites of insertions with the help of other protein domains and cellular proteins [24,25,26,27]. Non-LTR retrotransposons encode either an apurinic-like endonuclease or a restriction-like endonuclease, depending on their phylogenetic positions [2,28], and both endonucleases have been revealed to be involved in target-specific DNA cleavage [24,25,26,27,29]. The survival strategy of targeting multicopy genes can be so successful that R2, a non-LTR retrotransposon lineage found in diverse animals, has been maintaining its target specificity to the 28S ribosomal RNA (rRNA) genes for 850 million years [30,31].

In contrast to non-LTR retrotransposons, target sequence specificity in DNA transposons is less known. Several independent lineages of DNA transposons have been reported to show the target-specific integration. *Pokey* is a family of *piggyBac*-type DNA transposons encoding a DDD/E transposase and is specifically inserted in 28S rRNA genes [32]. Some families of *CryptonV*, a DNA transposon lineage encoding a YR, are known to be inserted specifically in microsatellites [33]. In prokaryotes, RNA-guided targeted integration of DNA transposons has been recently reported [34]. They encode a CRISPR effector Cas12k and a guide RNA to integrate themselves downstream of the DNA sequence annealed by the guide RNA. 

The *Dada* superfamily of DNA transposons is extraordinary DNA transposons many of which are inserted specifically inside of multicopy RNA genes [15]. *Dada* generates 6 or 7-bp target site duplications (TSDs) at both sides of insertion. The zebrafish genome harbors at least five families of *Dada* transposons. *Dada-U6_DR* is inserted in U6 snRNA genes, while *Dada-U1A_DR* and *Dada-U1B_DR* are in U1 snRNA genes. *Dada-tA_DR* is inserted in tRNA-Ala genes, and *Dada-tL_DR* is in tRNA-Leu genes. DNA transposons closely related to *Dada-U6_DR* are found in at least four animal phyla: Chordata, Arthropoda, Mollusca, and Annelida, and they are inserted at the same sites of U6 snRNA genes. Except for these *Dada-U6* families, *Dada-tA_OL*, found in the medaka *Oryzias latipes*, is the only example showing the same target specificity outside of the zebrafish genome. 

Here, vertebrate genomes are surveyed to characterize target-specific *Dada* families. Over 120 *Dada* families were characterized from the genomes of actinopterygian fish. The results show the wide presence of *Dada-U6*, *Dada-tA*, *Dada-tL*, and *Dada-U1* in actinopterygian fish. Besides that, newly characterized *Dada* families were found to be inserted in tRNA genes for Asp, Asn, Arg, Gly, Lys, Ser, Tyr, and Val, and 5S rRNA genes. The characterized *Dada* families could be grouped into 11 lineages based on their phylogenetic relationships and target specificities. A large number of *Dada* families with characterized target sequences would enhance the understanding of the targeted integration mechanism of *Dada* and help develop a tool for target-specific genetic transformation. 

## 2. Materials and Methods

### 2.1. Genome Survey

Censor [35] searches were performed against the genomes of various vertebrates with the protein sequence encoded by *Dada-U6_DR* [15]. Vertebrate genome sequences were downloaded from NCBI Assembly (https://www.ncbi.nlm.nih.gov/assembly (accessed on 13 January 2022)), GenomeArk of Vertebrate Genomes Project (https://vgp.github.io/ (accessed on 13 January 2022)), and China National GeneBank database (CNGBdb, https://db.cngb.org/cnsa/ (accessed on 13 January 2022)). The information of analyzed genome assemblies is shown in Table S1. Censor hits were extracted and clustered with BLASTCLUST 2.2.25 in the NCBI BLAST package with the thresholds at 75% length coverage and 75% sequence identity. The consensus sequence for each cluster was generated with the 50% majority rule applied with the help of homemade scripts. Censor [35] searches were performed with the consensus sequence of each cluster against the respective genome. Up to 10 Censor hits were extracted with 10,000-bp flanking sequences at both sides. Consensus sequences were regenerated to be elongated to reach small RNA gene sequences at both sides. The full-length consensus sequence of *Dada* was determined based on the comparison to the intact multicopy gene sequence. If BLASTCLUST did not generate any clusters of more than two genomic coordinates because of the low copy number of available sequences, Censor [35] search against the entire Repbase [13], which includes representative sequences of small RNA genes, was conducted with each hit genomic coordinate with 10,000-bp flanking sequences to find target multicopy genes; then, if multicopy genes were present at both sides of the hit genomic coordinate, the full-length sequence of *Dada* was determined based on the alignment between the intact multicopy gene sequence and the gene copy inserted by *Dada*. This full-length copy is considered as a representative of a *Dada* family. Only the *Dada* sequences flanked with target small RNA gene sequences were used for further analysis. In total, 121 *Dada* families were newly characterized from 51 fish species. All characterized consensus and representative sequences of *Dada* families are available as supplementary materials (Data S1) and are also submitted to Repbase (https://www.girinst.org/repbase/ (accessed on 13 January 2022)) [13]. Protein sequences encoded by the consensus and representative sequences of *Dada* families were predicted with the help of Softberry FGENESH [36], followed by manual corrections. 

### 2.2. Transcriptome Survey

TBLASTN was performed against all available transcriptome shotgun assemblies (TSA) of vertebrates with predicted protein sequences of *Dada-U6_DR*, *Dada-U6_DPu*, *Dada-U1A_DR*, *Dada-tL_DR*, *Dada-tA_DR*, *Dada-tA2_CaAu*, *Dada-tN_CaAu*, *Dada-tR_CaAu*, *Dada-tY_CaAu*, *Dada-5S-A_CaAu*, *Dada-tD_AnTe*, *Dada-tK_LaCr*, *Dada-tV_OJav*, and *Dada-U6B_PeFlu* as queries [15] (Data S1) on 22 July 2021, at the NCBI web server (https://blast.ncbi.nlm.nih.gov/Blast.cgi (accessed on 13 January 2022)). Up to 1000 hits were extracted from each TBLASTN run. Datasets were combined and duplicates were removed. The remaining 4348 DNA sequences were translated into protein sequences. Protein sequences that were shorter than 500 residues were removed from the analysis. The remaining 880 protein sequences and the *Dada* protein sequences used as queries were aligned with MAFFT with linsi option [37]. In total, 153 protein sequences of 530–668 residues, containing conserved motifs of *Dada* transposases were chosen for further analysis. 

### 2.3. Phylogenetic Analysis

Protein sequences predicted from the consensus or representative sequences for *Dada* families were aligned with the protein sequences derived from the transcriptome data. Any fragmented or partial protein sequences caused by the incorrect prediction of protein-coding sequences, or errors in sequencing or consensus-building were removed from the further analysis. Predicted protein sequences >90% identical to any of the protein sequences derived from transcriptome data were removed from the analysis. Maximum likelihood tree was generated at the PhyML 3.0 server (http://www.atgc-montpellier.fr/phyml/ (accessed on 7 November 2021)) with 100 bootstrapping supports [38]. The substitution model JTT + G + I + F was used based on the Akaike Information Criterion (AIC) [39]. The phylogenetic tree was rooted at the midpoint and visualized with FigTree v.1.4.3 (http://tree.bio.ed.ac.uk/software/figtree/ (accessed on 7 November 2021)).

## 3. Results

### 3.1. Wide Distribution and Various Target Specificities in Actinopterygian Dada Families

Vertebrate genomes were surveyed to characterize new *Dada* families of DNA transposons. The analysis could not detect any *Dada* families in tetrapods, coelacanths (*Latimeria chalumnae*), lungfish (*Neoceratodus forsteri*), elasmobranches, or agnathans (*Petromyzon marinus*, *Lethenteron camtschaticum*, *Eptatretus burgeri*). It is likely that in these lineages, *Dada* has been extinct or is present in very few copy numbers. 

Various *Dada* families were characterized from the genomes of actinopterygian fishes (Table 1 and Table S1). *Dada* families were found in the genomes of 19 fish orders. *Dada-U6* was found in seven orders (Clupeiformes, Cypriniformes, Salmoniformes, Gadiformes, Beloniformes, Perciformes, and Spariformes), *Dada-U1* in five orders (Cypriniformes, Anabantiformes, Beloniformes, Perciformes, and Tetraodontiformes), *Dada-tA* in five orders (Cypriniformes, Gadiformes, Beloniformes, Perciformes, and Spariformes) and Ambassidae of uncertain affinities, and *Dada-tL* in five orders (Cypriniformes, Carangiformes, Perciformes, Labriformes and Spariformes). 

**Figure 1:**
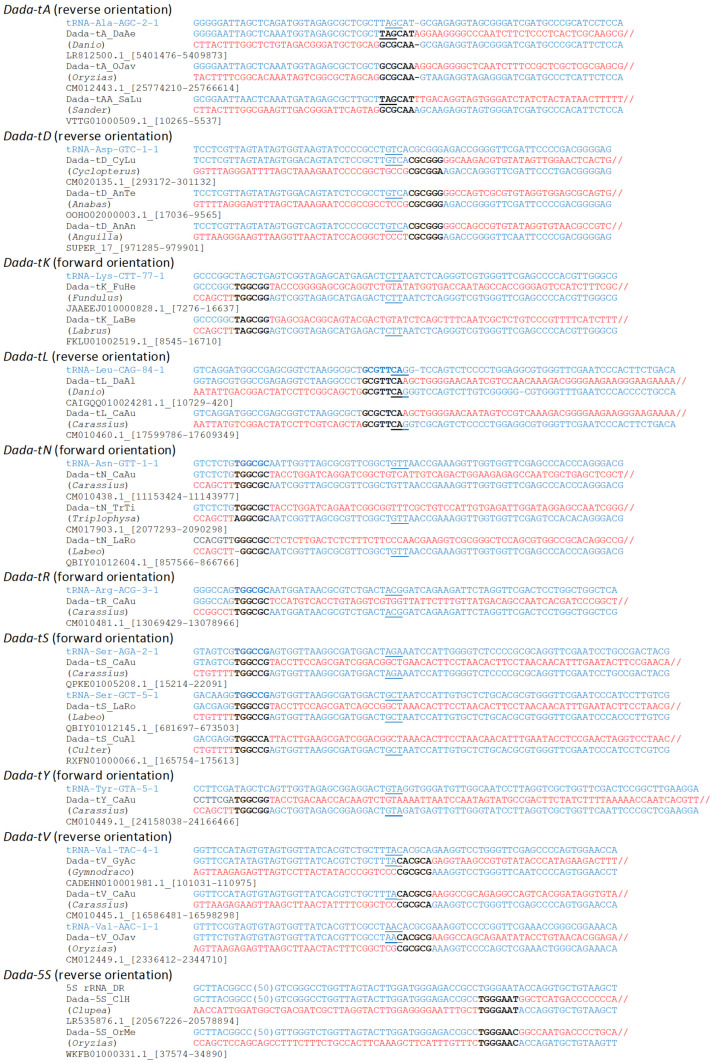
Target sequences of *Dada* families. Several representative insertions are shown with zebrafish tRNA genes. tRNA genes are based on GtRNA-DB (http://gtrnadb.ucsc.edu/ (accessed on 7 July 2021)). Genus names of the host organisms are shown in parentheses below the *Dada* family names. Accession numbers and locations are shown below the genus names. tRNA gene sequences are in blue, while *Dada* sequences are in red. TSDs are in bold. Anticodons are underlined.

Other types of tRNA genes were also revealed to be targeted by *Dada* families (Figure 1). Based on the types of the mainly targeted tRNA genes, *Dada* families were designated as *Dada-tD* targeting for tRNA-Asp, *Dada-tK* for tRNA-Lys, *Dada-tN* for tRNA-Asn, *Dada-tR* for tRNA-Arg, *Dada-tS* for tRNA-Ser, *Dada-tY* for tRNA-Tyr, and *Dada-tV* for tRNA-Val. In addition to these tRNA gene-targeting *Dada* families, *Dada* families targeting 5S rRNA genes were also found and designated as *Dada-5S*. 

Target specificity is not always strict enough to be inserted in only one type of tRNA gene (Figure S1). The genome of medaka Hd-rR contains five full-length copies of *Dada-tV_OL*. One copy is inserted in tRNA-Val-AAC and one in tRNA-Val-TAC. Two copies are inserted in tRNA-Gly-GCC. The other copy is inserted in a non-autonomous *hAT* family *hAT-N16_OL* with TSDs of CGCGCG. The genome of goldfish contains three copies of *Dada-tV_CaAu*. Two copies are inserted in tRNA-Ala-TGC and one is inserted in tRNA-Val-TAC. The genome of ploughfish contains four copies of *Dada-tV_GyAc*. Two copies are inserted in tRNA-Asp-GTC. One copy each is inserted in tRNA-Val-TAC and in tRNA-Val-CAC. Two copies of *Dada-tV_PeFlu* were found from the genome of European perch. They are inserted in tRNA-Asp-GTC and tRNA-Val-AAC respectively. In all of these cases, *Dada* copies are inserted in anticodon arm, despite the differences of target tRNA genes. *Dada-tY_CaAu* from goldfish is inserted in tRNA-Tyr-GTA (seven cases) and tRNA-Phe-GAA (two cases) (Figure S2). 

The previous study reported that the insertion of *Dada-tA_DR* replaces the flanking sequence of TAGCAT with GCGCAA [15]. Such replacement is also seen at integration sites of several *Dada* families, including the *Dada-tA* families from other fish species (Figure 1). Insertions of *Dada-tY_CaAu* replace TAGCTC with TGGCGG (Figure S2). Interestingly, this type of replacement is seen with insertions inside of either tRNA-Tyr or tRNA-Phe. Insertions of *Dada-tV* families seem to replace CACGCA with CGCGCG or CGCGCA, although these differences may reflect the divergence of tRNA genes themselves (Figure S1). 

### 3.2. Transcriptome Analysis

Homology search against TSA dataset available at NCBI BLAST website revealed the wider distribution of *Dada* transposons among actinopterygian fish (Table S2). Among them, 185 transcripts from 24 orders encode *Dada* transposases. Besides the 19 fish orders whose genomes retain *Dada* transposons, 8 orders (Acipenseriformes, Osteoglossiformes, Gymnotiformes, Esociformes, Callionymiformes, Pleuronectiformes, Atheriniformes, Centrarchiformes) are revealed to retain *Dada* in their genomes. A total of 23 transcripts show very high (>90%) sequence identity to the characterized *Dada* families. No *Dada* transcripts correspond to *Dada-tY* or *Dada-tK*. 

### 3.3. Phylogenetic Analysis

**Figure 2:**
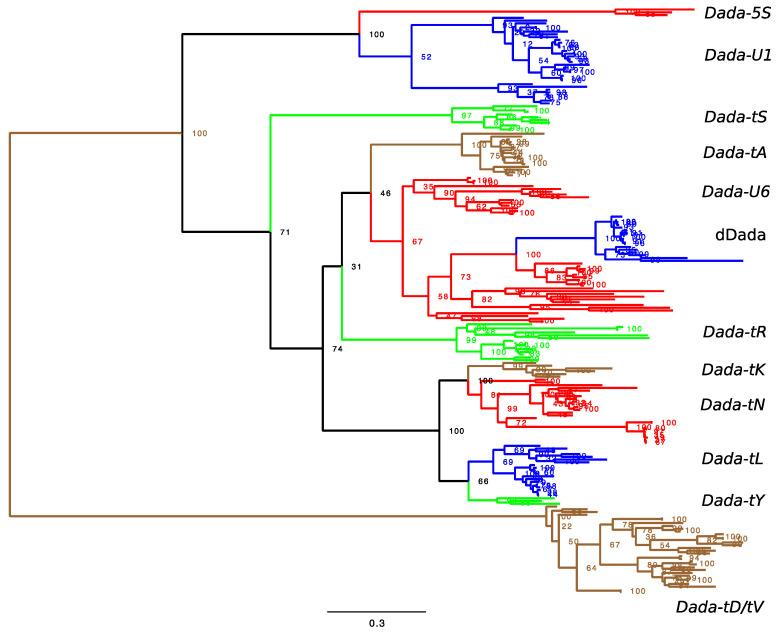
Phylogenetic tree of *Dada* transposases. Monophyletic or paraphyletic *Dada* lineages are colored based on the characterized target sequences. *Dada-tD* and *Dada-tV* are not well separated and thus shown as *Dada-tD*/*tV*. The complete phylogenetic tree with leaf names is available in Figure S3.

Phylogenetic analysis revealed several lineages inside of actinopterygian *Dada* families (Figure 2 and Figure S3). Here, a lineage that includes *Dada* families whose targets were confirmed is assumed to share the same target sequences. The most distant branch is composed of *Dada-tV* and *Dada-tD*. *Dada-tV* appears inside of *Dada-tD* in the phylogeny, but there is still a possibility that these two groups belong to the same group of weak target specificity. Indeed, *Dada-tV_GyAc* is inserted in both tRNA-Asp and tRNA-Val. Here, *Dada-tD* and *Dada-tV* are not yet determined as two independent groups and thus shown as *Dada-tD/tV*. 

The second branch is composed of *Dada-U1* and *Dada-5S*. Two *Dada-U1* families from the zebrafish genome, *Dada-U1A_DR* and *Dada-U1B_DR* are distant between each other and represent two independent sublineages inside of *Dada-U1* (Figure S3). The third branch is *Dada-tS*. *Dada-tK*, *Dada-tN*, *Dada-tL* and *Dada-tY* cluster together. The phylogenetic relationships among the remaining three groups, *Dada-tA*, *Dada-U6*, and *Dada-tR* are not well supported. 

### 3.4. dDada, Domesticated Dada-U6 in Cypriniformes

The transcript GFIL01015330.1 from zebrafish clustered together with other transcripts from various species belonging to Cypriniformes in the phylogeny (Figure S3). They show some unique features compared with other *Dada* transcripts. The two D residues in their DDE catalytic motif are often mutated. The conserved DxxH motif is also mutated to DxxQ. These mutated *Dada* transcripts are observed among fishes belonging to six families (Danionidae, Cyprinidae, Xenocyprididae, Tincidae, Gobionidae, and Leuciscidae) inside of the suborder Cyprinoidei (Table S3). The latter four families along with Acheilognathidae constitute a monophyletic lineage [40]. The phylogenetic relationships among these transcripts are consistent with the species phylogeny. Sequences from Cyprinidae, Danionidae, and the others (Xenocyprididae, Tincidae, and Leuciscidae) clustered separately (Figure S3). The zebrafish transcript GFIL01015330.1 corresponds to the gene id 100006560, which is located between S-phase kinase-associated protein 2 gene (gene id 563708) and Limb Development Membrane Protein 1 (LMBR1) domain-containing 2b gene (gene id 335257). It was confirmed that the transcripts similar to this zebrafish transcript are encoded at the orthologous loci in the genomes of goldfish (Cyprinidae) and fathead minnow (Leuciscidae), indicating the loci encoding these transcripts are conserved among all fishes in Cyprinoidei. 

The mutations of catalytic residues of transposase, orthologous loci among diverse fishes in Cyprinoidei, and the transcription observed suggest that they represent a gene derived from *Dada* transposon shared among Cyprinoidei. Here, it is designated as dDada for domesticated *Dada-U6*.

## 4. Discussion

### 4.1. Distributions of Target-Specific Dada Lineages

Here, the diversity of the *Dada* superfamily of DNA transposons in vertebrates was investigated. No target-specific *Dada* families were characterized outside of Actinopterygii in vertebrates. In Actinopterygii, 12 target specificities were observed (Table 1 and Table S1). Phylogenetic analysis showed that *Dada* families targeting the same target sequences cluster together, supporting that they have the common ancestor with the same target specificity (Figure 2 and Figure S3). Even though each target specificity was observed from diverse fish species, no genome contains all 12 target-specific *Dada* lineages (Table S1). The genome of the goldfish *C. auratus* contains the largest number of target-specific *Dada* lineages. The goldfish genome contains 10 lineages but does not have *Dada-tK* or *Dada-tD*. These two lineages were not observed from any genome of Cypriniformes. Still, goldfish lacks some of the *Dada* lineages orthologous to *Dada* families observed in other genomes in Cypriniformes. Goldfish do not have the family orthologous to *Dada-U1B_DR* or *Dada-U6_DR* from zebrafish (Figure S3). *Dada-U1A_CaAu* is orthologous to *Dada-U1A_DR*, another *Dada-U1* family from zebrafish. *Dada-U6_CaAu* is distant from *Dada-U6_DR* inside of the *Dada-U6* lineage. The lineage leading to goldfish has likely lost several *Dada* families. 

Each target-specific *Dada* lineage appears patchily distributed among Actinopterygii. Almost certainly, many *Dada* families have gone extinct in various lineages of Actinopterygii. It may also be explained by the horizontal transfer of *Dada* families between fishes, although in this study, no obvious horizontal transfer of *Dada* families was observed. On the other hand, some level of vertical transmission was supported (Figure S3). *Dada-U6_DR* from zebrafish has close relatives from other *Danio* species (*D. aesculapii*, and *D. choprai*). Their cluster is the sister lineage of *Dada-U6* families from two species of Cyprinidae (*Cyprinus carpio* and *Anabarilius grahami*). This relationship is consistent with the phylogenetic relationship of host organisms and suggests the vertical transmission of *Dada-U6* from the common ancestor of Cyprinidae and Danionidae.

### 4.2. Target Selection and Evolution of Dada

As reported in the cases of other target-specific TEs [18], the targets of *Dada* transposons are also selected based on their high copy number and their sequence conservation. The zebrafish genome encodes 8676 tRNAs, according to GtRNAdb (http://gtrnadb.ucsc.edu/GtRNAdb2/genomes/eukaryota/Dreri11/ (accessed on 7 July 2021)) [41]. Except for tRNA-Ala-AGC, all tRNA genes targeted by *Dada* in zebrafish have >100 copies. This indicates that each target-specific *Dada* family has plenty of potential target sequences, and also that the accumulation of tRNA genes disrupted by the *Dada* insertions would have little effect on the tRNA production and protein translation. 

When the integration sites of *Dada* transposons inside of tRNA genes are compared, there are two regions that accumulate the integration. One is around the anticodon loop, and this region is targeted by *Dada-tL*, *Dada-tA*, *Dada-tD*, and *Dada-tV* (Figure 1). Here, *Dada* is inserted in the opposite orientation compared to tRNA genes. *Dada-tV* and *Dada-tD* are closely related, but *Dada-tV*/*tD*, *Dada-tA* and *Dada-tL* are phylogenetically distant from one another (Figure 2 and Figure S3). Compared to the insertion sites of *Dada-tV* and *Dada-tD*, the site of *Dada-tA* is 5-bp upstream and the site of *Dada-tL* is 9-bp upstream (Figure 1). Their target specificities are likely selected independently to target the anticodon stem region. 

The other region to be targeted by *Dada* families is the boundary between the acceptor stem and the D-arm. Here, five types of *Dada* transposons (*Dada-tY*, *Dada-tK*, *Dada-tN*, *Dada-tR*, and *Dada-tS*) are inserted at the orthologous sites with putative 6-bp TSDs (Figure 1). This corresponds to the nucleotides 8–13 of mature tRNAs. Based on the phylogeny, *Dada-tY*, *Dada-tK*, *Dada-tN*, and *Dada-tR* are closely related (Figure 2 and Figure S3). Their target sequences are TAGCTC (*Dada-tY* and *Dada-tK*) or TGGCGC (*Dada-tN* and *Dada-tR*). *Dada-tS* is phylogenetically distant from the other four types of *Dada* transposons, and its targets are TGGCCG. 

According to GtRNAdb (http://gtrnadb.ucsc.edu/GtRNAdb2/genomes/eukaryota/ (accessed on 7 July 2021)) [41], in both zebrafish and medaka, six types of tRNA genes (Ala, Cys, Ile, Lys, Phe, and Tyr) contain the sequence TAGCTC at the nucleotides 8–13. Three types (Lys, Phe, and Tyr) among them are inserted by *Dada-tY* or *Dada-tK*. TGGCGC is seen in five types of tRNAs: Arg, Asn, Ile, Met, and Trp, and three types (Arg, Asn, and Met) of them are inserted by *Dada-tR* or *Dada-tN*. The target sequence of *Dada-tS* is TGGCCG, which is seen at the corresponding site in tRNA-Leu, in addition to tRNA-Ser. These facts indicate that these *Dada* lineages can target multiple types of tRNA genes. Target sequences of *Dada-tS*, *Dada-tK*, *Dada-tY* are followed by AG, while target sequences of *Dada-tR* and *Dada-tN* are followed by AA. 

**Figure 3:**
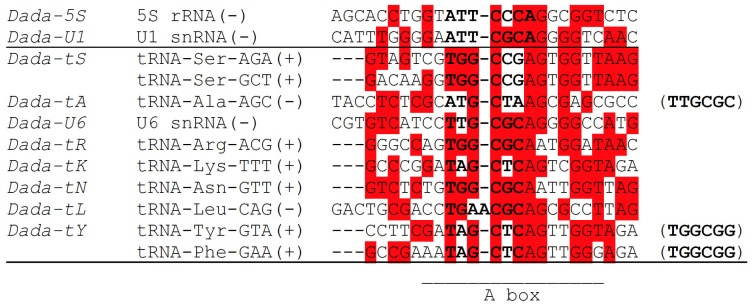
Sequence comparison of targets of *Dada*. From left, *Dada* family names, target RNA genes with directions (+, direct; − complementary), the nucleotide sequences around the insertion sites; if the TSDs are often replaced by another sequence, it is shown at the right in parentheses. The nucleotides corresponding to TSDs are in bold. The most frequent nucleotide is highlighted in red at each site. The A box of RNA polymerase III promoter found in tRNA genes is indicated.

The target sequences of *Dada-U1* and *Dada-5S* are similar to each other: ATTCGCAGGGGTC for *Dada-U1* and ATTCCCAGGCGGTC for *Dada-5S* (here not identical nucleotides are underlined) (Figure 3). As reported previously [15], the sequences around the insertion sites, CGCAGGGGCCA for *Dada-U6* and CGCAGGGGTCA for *Dada-U1* are almost identical to each other.

Sequence comparison of seven related *Dada* lineages targeting tRNA genes (*Dada-tS*, *Dada-tA*, *Dada-tR*, *Dada-tK*, *Dada-tN*, *Dada-tL*, and *Dada-tY*) and *Dada-U6*, which targets U6 snRNA genes, might shed light on the evolution of *Dada* with target changes (Figure 3). The targets of *Dada-tK*, *Dada-tN*, and *Dada-tY* are very similar to one another. *Dada-tL* is the sister lineage of *Dada-tY*, and targets TGAACGCAGCGCCTTAG, although the target is not orthologous to the target of *Dada-tY*. The target of *Dada-tL* shows the highest sequence resemblance to the target of *Dada-tN*, TGGCGCAATTGGTTAG. The primary sequence similarity indicates that *Dada* transposases recognize the nucleotide sequence itself. Besides, the higher sequence resemblance downstream from the insertion sites than upstream indicates the protein encoded by *Dada* is bound mainly downstream of the insertion sites. 

*Dada-U6* and *Dada-tA* could be the closest lineages. *Dada-U6* targets CTTGCGCAG in U6 snRNA genes. *Dada-tA* targets CATGCTAAG in tRNA-Ala. Often the insertion of *Dada-tA* replaces the target sequence ATGCTA with TTGCGC. This replacement can happen at only one side of *Dada-tA* insertion, implying that the transposition of *Dada* may not always generate TSDs. It can be speculated that *Dada-tA* was evolved from *Dada-U6* and the original flanking sequence TTGCGC has been transposed with the *Dada-tA* since then. It is noteworthy that the target sequences of *Dada-tY* are also often replaced by TGGCGG upon insertions. Such replacement occurs often at both sides of *Dada* insertions, and sometimes at one side (Figure S2). Similar to the case of *Dada-tA*, the sequence TGGCGG might be the ancestral target sequence for *Dada-tY*. tRNA-Ser contains TGGCGG at the orthologous site. tRNA-Gly also contains TGGCGG at the anticodon loop in its complementary strand. There might be or have been a *Dada* lineage targeting either of these target sequences. 

If the target sequence of the common ancestor of *Dada-U6* and *Dada-tA* was TTGCGC, the target of the common ancestor of *Dada-tK*, *Dada-tN*, *Dada-tL* and *Dada-tY* could be considered as TGGCGC, based on the sequence similarity. Although the phylogenetic placement of *Dada-tR* is uncertain, the ancestral target of all these lineages could be either TTGCGC or TGGCGC.

Remarkably, five *Dada* lineages targeting tRNA genes are inserted at the orthologous sites, nucleotides 8–13, inside of tRNA genes. *Dada* families targeting tRNA genes were also reported from the protist *Perkinsus marinus* [15]. Interestingly, they are also inserted at the orthologous sites in tRNA genes. The putative TSDs of *Dada-tIA_PMar*, *Dada-tIB_PMar*, *Dada-tY_PMar*, *Dada-tG_PMar* correspond to the nucleotides 8–13 of respective tRNA genes. They are TAGCTC followed by AG (*Dada-tIA_PMar*, *Dada-tIB_PMar*, and *Dada-tY_PMar*), or TAGTCT followed by AA (*Dada-tG_PMar*). *Dada-tY_PMar* and actinopterygian *Dada-tY* are not closely related. The nucleotides 8–13 are inside of A box of RNA polymerase III promoter. This means that the site targeted by many *Dada* families is under two different natural constraints; one is the promoter function for RNA polymerase III, and the other is the base-pairing for tRNA secondary structure. It can be speculated that *Dada* has long been maintained to keep their target specificity at the nucleotides 8–13 of tRNA genes with adapting their target recognition for the slight changes of target sequences. Some *Dada* families have acquired the ability to target other repetitive sequences, such as anticodon regions of tRNA genes, 5S rRNA genes, or snRNA genes, based on the similarity of primary nucleotide sequences.

## 5. Conclusions 

In this study, over 120 *Dada* families were characterized along with their target sequences. They are grouped into 11 lineages based on their phylogenetic relationships and their target specificities. Sequences targeted by phylogenetically related *Dada* lineages show sequence similarities, indicating that the target specificity of *Dada* is accomplished through the recognition of primary nucleotide sequences. This study provides a large number of protein sequences that recognize the same or similar target DNA sequences, which would help develop a system inserting a foreign DNA segment precisely at a specific locus in the genome.

## Figures and Tables

**Table 1:** Distribution of *Dada* families in Actinopterygii.

Order	Family ^1^	*Dada* Lineages with Confirmed Target Sequences											
		***U6***	***tA***	***tY***	***tL***	***tN***	***tK***	***tR***	***tS***	***U1***	***5S***	***th***	***tV***
Anguilliformes	Anguillidae (1)											+	
Clupeiformes	Clupeidae (1)										+		
	Engraulidae (1)	+							+			+	
Cypriniformes	Cyprinidae (8)	+	+	+	+	+		+	+	+	+		+
	Danionidae (8)	+	+		+	+				+			
	Nemacheilidae (1)	+	+			+							
Characiformes	Characidae (1)					+							
Siluriformes	Ictaluridae (1)								+				
	Pangasiidae (1)												+
Salmoniformes	Salmonidae (1)	+											
Gadiformes	Gadidae (1)	+	+						+				+
Scombriformes	Scombridae (1)											+	
Anabantiformes	Anabantidae (2)									+		+	
Carangiformes	Carangidae (1)				+	+							
Ovalentaria incertae sedis	Ambassidae (1)		+			+							
Mugiliformes	Mugilidae (1)					+							
Cichliformes	Cichlidae (2)											+	+
Beloniformes	Adrianichthyidae (3)	+	+					+		+	+		+
Cyprinodontiformes	Fundulidae (1)						+						
	Cyprinodontidae (1)								+				
	Poeciliidae (1)					+							
Perciformes	Percidae (5)	+	+		+	+			+	+		+	+
	Sciaenidae (1)		+				+						
	Cyclopteridae (1)											+	
	Nototheniidae (2)	+						+					+
	Gasterosteidae (1)	+											
Labriformes	Labridae (2)				+		+				+		
Spariformes	Sparidae (2)	+	+		+								
Tetraodontiformes	Tetraodontidae (1)									+			

^1^ Numbers in parentheses indicate the number of species from whose genome *Dada* was found.

## Data Availability

The data presented in this study are available as supplementary materials.

## Supplementary Materials

The following supporting information can be downloaded at: https://www.mdpi.com/article/10.3390/biology11020166/s1, Figure S1: Targets and flanking sequences of *Dada-tV_OL*, *Dada-tV_CaAu*, *Dada-tV_GyAc*, and *Dada-tV_PeFlu*; Figure S2: Targets and flanking sequences of *Dada-tY_CaAu*; Figure S3: Phylogenetic tree of *Dada* transposases; Table S1: *Dada* families found from Actinopterygii; Table S2: Transcripts of *Dada* DNA transposons found from TSA analysis; Table S3: dDada from Cypriniformes; Dataset S1: The nucleotide sequences of *Dada* families found in this study.
