# Supplementary material for: Diversity and Evolution of DNA Transposons Targeting Multicopy Small RNA Genes from Actinopterygian Fish
Source: Biology (Basel). 2022 Jan 20;11(2):166. doi: 10.3390/biology11020166 (PMC8869645; doi:10.3390/biology11020166)
Supplement: Supplementary file 1 [file biology-11-00166-s001.zip › Figures S1 and S2.pdf]

Dada-tV_OL

```
                     Stem         D-arm         Anticodon arm              T-arm        Stem
tRNA-Val-AAC-1-1     GTTTCCGTAGTGTAGTGGTTATCACGTTCGCCTAACACGCGAA--AGGTCCCCGGTTCGAAACCGGGCGGAAACA
Dada-tV_OL           --TTCCGTAGTGTAGTGGTTATCACGTTCGCCTAACACGCGAAGGGGCCAGCCGTATATACCTGTAACACGGAACAGCCAATCAAATTGCAGCTA//
                     --AGTTAAGAGAGTTAAGCTTAACCATTTTCGGCTCCCGCGCGGGAGGTCCCCGGTTCGAAACCGGGCGGAAACACTTATATTTTCTTTTTTTTATT
CM014820.1_[3380803-3387985]
                     Stem         D-arm         Anticodon arm           T-arm        Stem
tRNA-Val-TAC-8-1     GTTTCTGTAGTGTAGTGGTTATCACGTTCGCCTTACACGCGAAAGGTCCCCAGTTCAAAACTGGGCGGAAACA
Dada-tV_OL           GTTTCTGTAGTGTAGTGGTTATCACGTTCGTCTTACACACGAAAGGGGGCCAGCCGTATATACCTGTAACACGGAACAGCCAATCAAATTGCAGC//
                     AGTTAAGAGAGTTAAGCTTAACCATTTTCGGCTCCCGCACGAAAGGACCCCAGCTCGAACCTGGGCAGAGACAGGACTTTCCCTTCTTAAATTGAAG
CM014819.1_[31653287-31660457]
                     Stem         D-arm         Anticodon arm           T-arm        Stem
tRNA-Gly-GCC-1-1     -GCATTGGTGGTTCAGTGGTAGAATTCTCGCCTGCCATGCGGGAGACCCGGGTTCGATTCCCGGCCAATGCA
Dada-tV_OL           TGCATTGGTGGTTCAGTGGTAGAATTCTCGCCTGCCACGCGAAGGCCAGCCGTATATACCTGTAACACGGAACAGCCAATCAAATTGCAGCTACT//
                     AGTTAAGAGAGTTAAGCTTAACCATTTTCGGCTCCCGCGCGGGAGGCCCGGGTTCGATTCCCGGCCAATGCATGTATTTATTTTTCTCTCGATCCTG
CM014819.1_[2908526-2901344]
Dada-tV_OL           TGCATTGGTGGTTCAGTGGTAGAATTCTCGCCTGCCACGCGGGGGCCAGCCGTATATACCTGTAACACGGAACAGCCAATCAAATTGCAGCTACT//
                     AGTTAAGAGAGTTAAGCTTAACCATTTTCGGCTCCCGCGCGGGAGGCCCGGGTTCGATTCCCGGCCAATGCAAGTATCTCTTTTTCTCAACCCTCAA
CM014819.1_[4779104-4771935]

hAT-N16_OL           AGGTGTAACGCCTCAGGCACGGCAAATGCCGCCCCCTATGAAGTGCCGCCCTGGGCGACCGCCCACATCGCCCATATCAAAAACCGCCACTG
Dada-tV_OL           AGGTGTAACGCCTCAGGCACGGCAAATGCCGCCCCCTCGCGCGGGCCAGCCGTATATACCTGTAACACGGAACAGCCAATCAAATTGCAGCT//
                     AGTTAAGAGAGTTAAGCTTAACCATTTTCGGCTCCCGCGCGCG--------------ACCGCCCACATCGCCCATATCAAAAACCGCCACTG
CM014830.1_[17201095-17193896]
```

Dada-tV_CaAu

```
                     Stem         D-arm         Anticodon arm              T-arm        Stem
tRNA-Ala-TGC-15-1    -GGGGATGTAGCTCAGTGGTAGAGCGCATGCTTTGCG--TGTATGAGGTCCTGGGTTCAATCCCCAGCATCTCCA
Dada-tV_CaAu         TGGGGATGTAGCTCAGTGGTAGAGCGCATGCTTTGCACGCGGGGGCCAGTCACGGATAGGTGTAGAGCGCTAACTAGCCAATAGGAACGAAGG//
                     GTTAAGAGAAGTTAAGCTTAACTATTTTCGGCTCCCGCGTGTATGAGGTCCTGGGTTCAATCCCCAGCATCTCCAAACACTGTTTGCTGAGTTTAAG
QPKE01005959.1_[12699-862]
Dada-tV_CaAu         TGGGGATGTAGCTCAGTGGTAGAGCGCATGCTTTGCACGCGGGGGCCAGTCACGGATAGGTGTAGAGCGCTAACTAGCCAATAGGAACGAAGGAA//
                     GTTAAGAGAAGTTAAGCTTAACTATTTTCGGCTCCCGCGTGTATGAGGTCCTGGGTTCAATCCCCAGCATCTCCAAACACTGTTTGCTGAGTTTAAG
QPKE01003285.1_[32671-20852]
                     Stem         D-arm         Anticodon arm              T-arm        Stem
tRNA-Val-TAC-4-1     GGTTCCATAGTGTAGTGGTTATCACGTCTGCTTTACACGCAGAAGGTCCTGGGTTCGAGCCCCAGTGGAACCA
Dada-tV_CaAu         GGTTCCATAGTGTAGTGGTTATCACGTCTGCTTTACACGCGAAGGCCGCAGAGGCCAGTCACGGATAGGTGTAGAGCGCTA//
                     GTTAAGAGAAGTTAAGCTTAACTATTTTCGGCTCCCGCGCAGAAGGTCCTGGGTTCGAGCCCCAGTGGAACCAAGCGTTTTTAGAAAAGATTC
CM010445.1_[16586481-16598298]
```

Dada-tV_GyAc

```
                     Stem         D-arm         Anticodon arm               T-arm        Stem
tRNA-Asp-GTC-1-1     TCCTCGTTAGTATAGTGGTAAGTATCCCGCCTGTCACGCGGG-AGACCGGGGTTCGATTCCCCGACGGGGAG
Dada-tV_GyAc         TCCTCGTTAGTATAGTGGTCAGTATCCCCGCCTGTCACGCGGGGGTAAGCCGTGTATACCCATAGAAGACTTTCTATTGGCCA//
                     AGTTAAGAGAGTTAGTCCTTACTATACCCGGTCCCCGCGCGGG-AGACCGGGGTTCAATTCCCCGACGGGGAGAGTACGCACTTTTTTGTTGATG
CADEHN010000867.1_[122816-115008]
                     TCCTCGTTAGTATAGTGGTCAGTATCCCCGCCTGTCACCCCGAGGTAAGCCGTGTATACCCATAGAAGACTTTCTATTGGCCAG//
                     AGTTAAGAGAGTTAGTCCTTACTATACCCGGTCCCCGCCGCGGGAGACCGGGGTTCAATTCCCCGACGGGGAGGaaatactttattat
CADEHN010001009.1_[31883-39662]
                     Stem         D-arm         Anticodon arm              T-arm        Stem
tRNA-Val-TAC-4-1     GGTTCCATAGTGTAGTGGTTATCACGTCTGCTTTACACGCAGAAGGTCCTGGGTTCGAGCCCCAGTGGAACCA
Dada-tV_GyAc         GGTTCCATATAGTAGTGGTTATCACGTCTGCTTTACACGCAGAGGTAAGCCGTGTATACCCATAGAAGACTTTCTATTGGCCAG//
                     AGTTAAGAGAGTTAGTCCTTACTATACCCGGTCCCCGCGCGAAAGGTCCTGGGTTCAATCCCCAGTGGAACCTGATGTCTGTCTTGTTGT
CADEHN010001981.1_[101031-110975]
                     Stem         D-arm         Anticodon arm              T-arm        Stem
tRNA-Val-CAC-1-1     GTTTCCGTAGTGTAGTGGTTATCACGTTCGCCTCACACGCGAAAGGTCCCCGGTTCGAAACCGGGCGGAAACA
Dada-tV_GyAc         GTTTCCGTAGTGTAGTGGTTATCACGTTCGCCTCACACGCGAAGGTAAGCCGTGTATACCCATAGAAGACTTTCTATTGGCC//
                     AGTTAAGAGAGTTAGTCCTTACTATACCCGGTCCCCGCGCGAAAGGTCCCCGGTTCGAGACCGGGCGGAAACACATTatgtcttgtttgctt
CADEHN010000507.1_[133765-141575]
```

Dada-tV_PeFlu

```
                     Stem         D-arm         Anticodon arm              T-arm        Stem
tRNA-Asp-GTC-1-1     TCCTCGTTAGTATAGTGGTAAGTATCCCGCCTGTCACGCGGGAGACCGGGGTTCGATTCCCCGACGGGGAG
Dada-tV_PeFlu        TCCTCGTTAGTATAGTGGACAGTATCTCCGCCTGTCACGCGGAGGCCAGTCGCGTATAGGTGTAGAAGACTTTTTCG//
                     GTTAAGAGAATTAAGCCCTACTATTCACGGCTCTGGCCGCGGAAGACCGGGGTTCGATTCCCCGACGGGGAGAATATTCTACCTCTTAATTATT
CM020910.1_[3366611-3358464]
                     Stem         D-arm         Anticodon arm              T-arm        Stem
tRNA-Val-AAC-1-1     GTTTCCGTAGTGTAGTGGTTATCACGTTCGCCTAACACGCGAAAGGTCCCCGGTTCGAAACCGGGCGGAAACA
Dada-tV_PeFlu        GTTTCCGTAGTGTAGTGGTTATCACGTTCGCCTAACACGCGGAGGCCAGTCGCGTATAGGTGTAGAAGACTTTTTCG//
                     GTTAAGAGAATTAAGCCCTACTATTCACGGCTCCGGGCGGAAACATTGTTGGCTTTTCCCTACGGTAGGAAGCTCCCGTGTTTGGTGCAAAGT
CM020924.1_[3435000-3443166]
```

Figure S1. Targets and flanking sequences of *Dada-tV_OL, Dada-tV_CaAu, Dada-tV_GyAc*, and *Dada-tV_PeFlu*. Zebrafish tRNA genes are based on GtRNA-DB (http://gtrnadb.ucsc.edu/). Accession numbers and locations are shown below the *Dada* family name. Sequences of base pairing in the tRNA secondary structure are highlighted in the same color. tRNA gene sequences are in blue, while *Dada* sequences are in red. TSDs are in bold. Anticodons are underlined.

```
                     Stem           D-arm       Anticodon arm (intron)                    T-arm       Stem
tRNA-Tyr-GTA-5-1   CCTTCGA-TAGCTCAGTTGGTAGAGCGGAGGACTGTAGgtgggatgttggcaATCCTTAGGTCGCTGGTTCGACTCCGGCTTGAAGGA
Dada-tY_CaAu       CCTTCGA-TGGCGGTACCTGACAACCACAAGTCTGTAAAATTAATCCAATAGTATGCCGACTTCTATCTTTTAAAAACCAATCA//
                   CCCAGCTTTGGCGGAGCTGGTAGAGCGGAGGACTGTAGATGAGTTGTTGGGTATCCTTAGGTCGCTGGTTCAATTCCCGCTCGAAGGA
CM010449.1_[24158038-24166466]
                   CCTTCGA-TGGCGGTACCTGACAACCACAAGTCTGTAAAATTAATCCAATAGTATGCCGACTTCTATCTTTTAAAAACCAATCA//
                   CCCAGCTTTAGCTCAGCTGGTAGAGCGGAGGACTGTAGATGAGTTGTTGGGTATCCTTAGGTCGCTGGTTCAATTCCGGCTCGAAGGA
QPKE01004703.1_[17626-9309]
                   CCTTCGA-TGGCGGTACCTGACAACCACAAGTCTGTAAAATTAATCCAATAGTATGCCGACTTCTATCTTTTAAAAACCAATCA//
                   CCCAGCTTTGGCGGAGCTGGTAGAGCGGAGGACTGTAGATGAGTTGTTGGGTATCCTTAGGTCGCTGGTTCAATTCCGGCTCGAAGGA
QPKE01008127.1_[3793-12211]
                   CCTTCGA-TGGCGGTACCTGACAACCACAAGTCTGTAAAATTAATCCAATAGTATGCCGACTTCTATCTTTTGAAAACCAATCA//
                   CCCAGCTTTGGCGGAGCTGGTAGAGCGGATGACTGTAGATGAGTTGTTGGGTATCCTTAGGTCGCTGGTTCAATTCCGGCTCGAAGGA
CM010449.1_[24118929-24127125] (This insertion has a duplication of the 5’ ~300 bp sequence)
tRNA-Tyr-GTA-2-1   CCTTCGA-TAGCTCAGTTGGTAGAGCGGAGGACTGTAGgtgggatgatggca-ATCCTTAGGTCGCTGGTTCGACTCCGGCTCGAAGGA
                   CCTTCGA-TGGCGGTACCTGACAACCACAAGTCTGTAAAATTAATCCAATAGTATGCCGACTTCTATCTTTTAAAAACCAATCA//
                   CCCAGCTTTGGCGGAGCTGGTAGAGCGGAGGACTGTAGGTTGGAGTGTTGGCCATCCTTAGGTCGCTGGTTCAACTCCGGCTCGAAGGA
QPKE01004295.1_[22555-14199]
                   CCTTCGA-TGGCGGTACCTGACAACCACAAGTCTGTAAAATTAATCCAATAGTATGCCGACTTCTATCTTTTAAAAACCAATCA//
                   CCCAGCTTTGGCGGAGCTGGTAGAGCGGAGGACTGTAGGTTGGAGTGTTGGCAATCCTTAGGTCGCTGGTTCGACTCCGGCTCGAAGGA
QPKE01005872.1_[9228-909]
tRNA-Tyr-GTA-1-2   CCTTCGA-TAGCTCAGTTGGTAGAGCGGAGGACTGTAGcggattatcactgaaATCCTTAGGTCGCTGGTTCGAATCCGGCTCGAAGGA
                   CCTTCGATTGGCGGTACCTGACAACCACAAGTCTGTAAAATTAATCCAATAGTATGCCGACTTCTATCTTTTAAAAACCAATCA//
                   CCCAGCTTTGGCGGAGTTGGTAGAGCGGAGGACTGTAGCGGA--ATCACTGTAATCCTTAGGTCGCTGGTTCGAATCCGGCTCGAAGGA
QPKE01004906.1_[62158-53971]

                     Stem           D-arm       Anticodon arm           T-arm       Stem
tRNA-Phe-GAA-1-1   -GCCGAAATAGCTCAGTTGGGAGAGCGTTAGACTGAAGATCTAAAGGTCCCTGGTTCGATCCCGGGTTTCGGCA
                   GGCCGAAATGGCGGTACCTGACAACCACAAGTCTGTAAAATTAATCCAATAGTATGCCGACTTCTATCTTTTAAAAACCAATCA//
                   CCCAGCTTTAGCTCAGTTGGGAGAGCGTTAGACTGAAGATCTAAAGGTCCCTGGTTCGATCCCGGGTTTCGGCA
QPKE01003480.1_[8408-16882]
                   GGCCGAAATGGCGGTACCTGACAACCACAAGTCTGTAAAATTAATCCAATAGTATGCCGACTTCTATCTTTTAAAAACCAATCA//
                   CCCAGCTTTAGCTCAGTTGGGAGAGCGTTAGACTGAAGATCTAAAGGTCCCTGGTTCGATCCCGGGTTTTGGCA
QPKE01008138.1_[17460-25669]

                   GCTCCCCCCAGTGCGGTACCTGACAACCACAAGTCTGTAAAATTAATCCAATAGTATGCCGACTTCTATCTTTTAAAAACCAATCA//
                   CCCAGCTTCAGTGCTGACGCCCGTCCATCTGACCGCGTCTTGACGCATGCTGTTTTGGAGTCACAACAGCAAATCGTTATGGCCATCGGAGAAATTA
CM010472.1_[10857147-10848814]
```

Figure S2. Targets and flanking sequences of *Dada-tY_CaAu*. Zebrafish tRNA genes are based on GtRNA-DB (http://gtrnadb.ucsc.edu/). Accession numbers and locations are shown below the *Dada* family name. Sequences of base pairing in the tRNA secondary structure are highlighted in the same color. tRNA gene sequences are in blue, while *Dada* sequences are in red. TSDs are in bold. Anticodons are underlined.
