# Supplementary figures and images for: Diversity and Evolution of DNA Transposons Targeting Multicopy Small RNA Genes from Actinopterygian Fish
Source: Biology (Basel). 2022 Jan 20;11(2):166. doi: 10.3390/biology11020166 (PMC8869645; doi:10.3390/biology11020166)

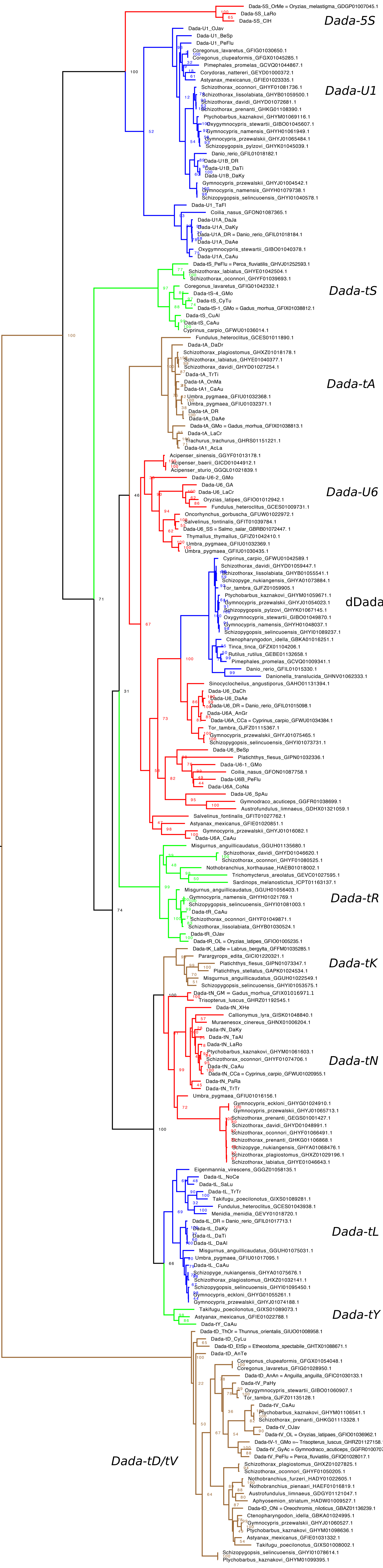

Supplement: Supplementary file 1 [file biology-11-00166-s001.zip › Figure S3.pdf]
